# Evaluation of two independent protocols for the extraction of DNA and RNA from different tissues of sea cucumber *Isostichopus badionotus*

**DOI:** 10.1016/j.mex.2019.07.010

**Published:** 2019-07-12

**Authors:** Carlos Puch-Hau, Itzel A. Sánchez-Tapia, Victoria Patiño-Suárez, Miguel A. Olvera-Novoa, Mariel Gullian Klanian, Mercedes Quintanilla-Mena, Omar Zapata-Pérez

**Affiliations:** a Centro de Investigación y de Estudios Avanzados del Instituto Politécnico Nacional (CINVESTAV), Departamento de Recursos del Mar, Unidad Mérida, Km. 6 Antigua Carretera a Progreso, Apdo. Postal 73-Cordemex, 97310, Mérida, Yucatán, Mexico; b Universidad Marista de Mérida, Periférico Norte Tablaje Catastral 13941, Carretera Mérida-Progreso, P.O. Box 97300, Mérida, Yucatán, Mexico

**Keywords:** *I. badionotus* DNA and RNA isolation, Holothuroid, Molecular biology, Genetics, Nucleic acid

## Abstract

*Isostichopus badionotus* is a sea cucumber species of great ecological and economic relevance for Mexico and Central American and Caribbean countries; however, the protocols for the extraction of the nucleic acids have not yet been published. In this study, we describe the first protocols to obtain DNA and RNA from different tissues of *I. badionotus,* which include the respiratory tree, gonad, longitudinal muscle bands, anterior intestine and cloaca. The extraction of high-quality DNA was performed using the DNeasy Blood & Tissue kit (Qiagen, Valencia, CA, USA) with minor modifications in different points of the protocol.

Concerning the RNA, the method of TRIzol was used. This method is particularly advantageous in situations where cells or tissues are enriched for endogenous RNases or when the separation of cytoplasmic RNA from nuclear RNA is impractical. The methodologies used in this study allowed us to obtain DNA and RNA of high quality and integrity in the different tissues of *I. badionotus,* which will be the basis for future genomic and transcriptomic studies.

• The successful extraction of DNA and RNA was achieved in the different tissues of *I. badionotus*.
• The concentrations of DNA and RNA obtained were adequate for a diversity of analyses at a molecular level.

## Graphical abstract

**Figure:**
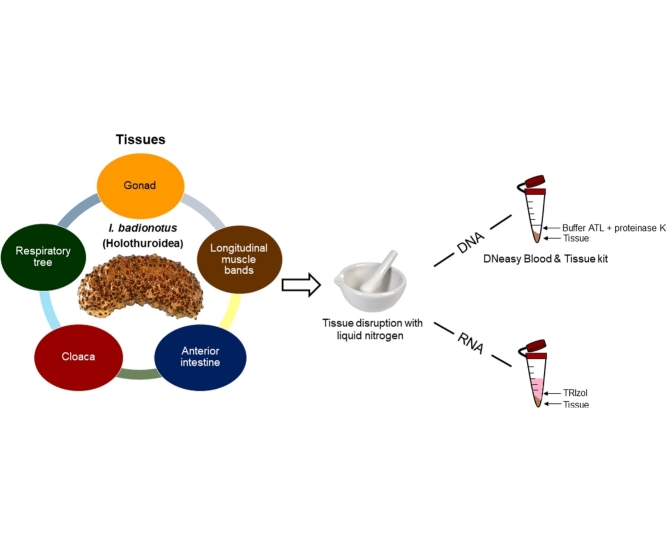

**Specifications Table**

**Table:** 

Subject Area:	Biochemistry, Genetics and Molecular Biology
More specific subject area:	Marine molecular biology
Method name:	*I. badionotus* DNA and RNA isolation
Name and reference of original method:	P. Chomczynski, N. Sacchi, Single-step method of RNA isolation by acid guanidinium thiocyanate-phenol-chloroform extraction, Anal Biochem 162 (1987) 156-159. http://doi.org/10.1006/abio.1987.9999.
Resource availability:	This study was partially supported by the Project Fomix-Conacyt YUC-2011-C09-169961 “Aportaciones al conocimiento para el desarrollo tecnológico del cultivo de pepino de mar en Yucatán”

## Method details

### Background

The sea cucumber *I. badionotus* (deuterostome of phylum Echinodermata), is a resource of great economic importance for the south-east region of the Gulf of Mexico and Central American and Caribbean countries. The demand in the international market, mainly from the Asian countries, has increased, with prices per kg of the dry salty product ranging from USD 203 to 402 in Hong Kong China SAR (Special Administrative Region) [1]. Due to its high price in the market and the threat to wild populations in relation to overfishing, various efforts are being carried out in order to cultivate this organism, with significant progress achieved in its growth and nutrition [2,3]. Now is the moment to initiate genetic studies in cultured organisms, therefore, the standardization of methodologies to obtain DNA and RNA of high quality and integrity is required, in order to carry out breeding studies based on genetics, or studies of physiological-metabolic expression, among others. The primary objective of this study was the evaluation of two independent protocols for the isolation of DNA and RNA from the different tissues of *I. badionotus*. Obtaining DNA and RNA from different parts has important implications for studies of methylation and tissue-specific gene expression [4,5]. For the extraction of DNA, the commercial DNeasy Blood & Tissue kit (Qiagen, Valencia, CA, USA) was used. The DNA obtained from this method has been used successfully in diverse studies of epigenetics [6], microbiome analysis [7] and population genetics [8]. Concerning the RNA, the method of TRIzol was used. This method is particularly advantageous in situations where cells or tissues are enriched for endogenous RNases or when the separation of cytoplasmic RNA from nuclear RNA is impractical [9]. Many studies at the transcriptomic level in several marine species have been generated using the RNA extracted with this methodology [[10], [11], [12]].

### Organisms

Adults sea cucumber *I. badionotus* were collected from the wild population of the coast of Dzilam de Bravo, in the Yucatan Peninsula, Mexico. Individuals were caught in sandy areas at depths up to 5 meters (m), with bottom and surface temperatures of 24.5 °C and 26.5 °C, respectively, using the Air-Line hookah diving method. Collected organisms were transported in coolers with natural seawater at 24 °C to the marine station of CINVESTAV located in Telchac Puerto, Yucatan, Mexico, where they were gradually acclimatized to laboratory conditions: 25 °C temperature, 34‰ salinity, 4.5 mg/L dissolved oxygen and 13:11 hours (h) photoperiod (light/darkness, respectively). Organisms were kept in two fiberglass tanks of 1000 L capacity, with a water column depth of 40 cm, and 2.8 m^2^ of a bottom surface area. These tanks were connected to a recirculating aquaculture system with sand filter, biological filter (Nitrobacter), skimmer, and UV sterilization for water quality control.

In order to isolate the DNA and RNA of the species, a total of four adult organisms were desensitization with ice-immersion baths for 3 h and then dissected to extract the main internal organs: left and right respiratory trees, gonad, longitudinal muscle bands, anterior intestine, and cloaca. A portion of 5 g of each tissue was immediately frozen in liquid nitrogen, and stored at −80 °C for 24 h before the analysis.

### DNA extraction

#### Reagents

Molecular Biology Grade Ethanol (Sigma).

#### Protocol

For the extraction of the DNA we used the DNeasy Blood & Tissue kit (Qiagen, Valencia, CA, USA) with minor modifications.

1 Approximately 25 mg of tissue were powdered in presence of liquid nitrogen and placed in a 1.5 mL microcentrifuge tube.
2 Subsequently, 180 μL Buffer ATL were added and homogenized.
3 Immediately, 20 μL of proteinase K were added and mixed by vortexing.
4 The samples were incubated at 56 °C for 2 h until the sample was completely lysed. During the incubation, we mixed the samples every 15 min, approximately.
5 After the incubation, the samples were vortexed for 15 s and 200 μL of Buffer AL were added. Subsequently, they were mixed vigorously for 10 s, in order to avoid the formation of precipitates.
6 Two hundred microliter of ETOH at 100% were added and mixed for 10 s.
7 Using a pipette, the mixture was deposited in a DNeasy Mini spin column (we avoid the transport of precipitates so as not to obstruct the column) and placed in a 2 mL collection tube.
8 These were then centrifuged at 8000 rpm for 3 min (or until the entire sample has passed through the column). The flow-through and collection tube were discarded.
9 The columns were placed in a new 2 mL collecting tube and 500 μL buffer AW1 were added in the center of the column (taking care not to touch the filter in order to avoid perforation). The samples were centrifuged at 8000 rpm for 1 min. The flow-through and collection tube were discarded.
10 Once again the columns were placed in a 2 mL collection tube and 500 μL of Buffer AW2 were added in the center of the column. The samples were centrifuged at 13, 000 rpm for 3 min. The flow-through and collection tube were discarded.
11 In addition to what the kit protocol indicates, the samples were centrifuged for 1 min at 13,000 rpm, with the aim of eliminating the residues of Buffer AW2 contained in the column. The flow-through and collection tube were discarded.
12 Each column was transferred to a new 1.5 mL tube and 20 μL of ultrapure water were added in the center of the column to elute the DNA. The columns were incubated for five minutes at room temperature (15–25 °C) and subsequently centrifuged for 1 min at 8000 rpm.
13 In order to increase DNA concentration, the flow-through was recovered and passed through the column once again. The samples were stored at −20 °C.
14 The concentration and integrity of total DNA were determined using a Nanodrop 1000 (Thermo Scientific, USA) and 1% agarose gel electrophoresis.

#### Treatment with RNase

1 0.2 μL of RNase *(Ribonucleasa A sigma)* was added to each one of the samples.
2 Subsequently, these were incubated for 1 h at 37 °C and stored at −20 °C.

#### Notes on the protocol

The whole process of DNA extraction was carried out at room temperature.

### RNA extraction

#### Reagents

1 Trizol™ Reagent (Invitrogen, Carlsbad, CA, USA).
2 Chloroform (Sigma).
3 Molecular Biology Grade Ethanol (Sigma).

#### Protocol

The extraction of RNA was performed using the method of TRIzol reported by Chomczynsky [13].

1 Approximately 100 mg of tissue were powdered in presence of liquid nitrogen, and placed in a 1.5 mL microcentrifuge cold tube.
2 Immediately, 1 mL of TRIzol™ Reagent was added to the sample and vigorously vortexed until it was completely homogenized.
3 The samples were then incubated over night at 4 °C.
4 Subsequently, they were centrifuged at 13,000 rpm for 10 min at 4 °C. The samples, in which separation of phases was not observed, were centrifuged for an additional 10 min.
5 The supernatant was transferred to a new tube and 200 μL of cold chloroform were added. The samples were vigorously agitated for 15 s and incubated for 2–3 min at room temperature.
6 The samples were then centrifuged at 13,000 rpm for 15 min at 4 °C and the supernatant was recovered and placed in a new 1.5 ml tube.
7 Five hundred μL of cold isopropanol were added to the supernatant and mixed by inversion. The samples were incubated for two hours at −20 °C.
8 Subsequently, they were centrifuged at 13,000 rpm for 10 min at 4 °C to precipitate the total RNA. The supernatant was discarded and the RNA pellet was washed with 1 mL of cold ETOH at 75%.
9 The samples were then washed again with 1 mL of ETOH at 100% and centrifuged at 13,000 rpm for 10 min at 4 °C.
10 The RNA pellet was allowed to dry under cold conditions for approximately 10–15 min, after which they were resuspended in 20–50 μL of water with RNase inhibitors and subsequently stored at −80 °C.
11 The concentration and integrity of total RNA were determined using a Nanodrop 1000 (Thermo Scientific, USA) and 2% agarose gel electrophoresis.

#### Notes on the protocol

1 The tissues extracted from the organism were frozen immediately in liquid nitrogen in order to avoid RNA degradation.
2 The material used for the process of RNA extraction was previously sterilized.
3 All of the reactives used for RNA extraction were previously cooled to 4 °C.
4 The process of RNA extraction was carried out under cold conditions, always avoiding thawing of the tissues.
5 The water with RNase inhibitors was prepared by adding 5 μL of RNasin® Ribonuclease Inhibitors (Promega, Madison, Wisconsin, USA) to 2 mL of nuclease free water.

#### Conventional PCR assays

In order to validate the DNA and RNA quality, we amplified a fragment corresponding to 18S rRNA gene.

1 The cDNA was synthesized using random hexamer primers (Sigma-Aldrich) and 500 ng of RNA in conjunction with ImProm-II™ Reverse transcriptase (Promega, USA) according to the manufacturer’s protocol.
2 The polymerase chain reactions (PCR) were performed in 20 μL volumes with the following concentrations: 1 μL sample of DNA (50 ng) and 2 μL of cDNA, 4 μL of 5X Phire Reaction Buffer, 1 μL of each primer to 10 μM (forward: TTCCGATAACGAACGAGACTCCG and reverse: GGACATCTAAGGGCATCACAGACC), 1 μL of the dNTPs mix to 10 mM (Promega, USA) and 0.4 μL of Phire Hot Start II DNA Polymerase (Thermo Scientific, USA).
3 Thermal cycling conditions were established as follows: 98 °C for 30 s (initial denaturation); followed by 40 cycles of 98 °C for 5 s, with an annealing temperature of 40 °C for 5 s and 72 °C for 10 s; the final elongation step was set at 72 °C for 10 min.

### Method validation

**Fig. 1:**
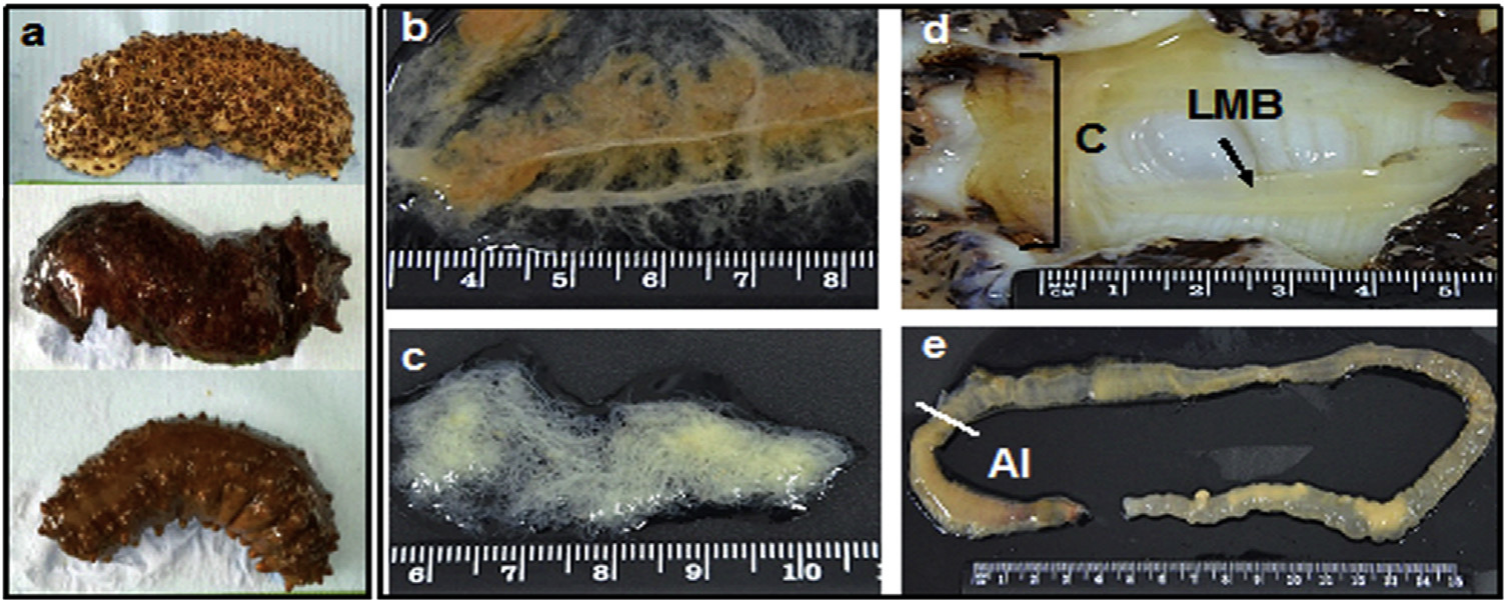
Specimens of *I. badionotus* dissected for the extraction of DNA and RNA. (a) Color variants, (b) Respiratory Tree, (c) Gonad, (d) Cloaca and Longitudinal Muscle Bands (LMB) and (e) Anterior Intestine (AI).

**Fig. 2:**
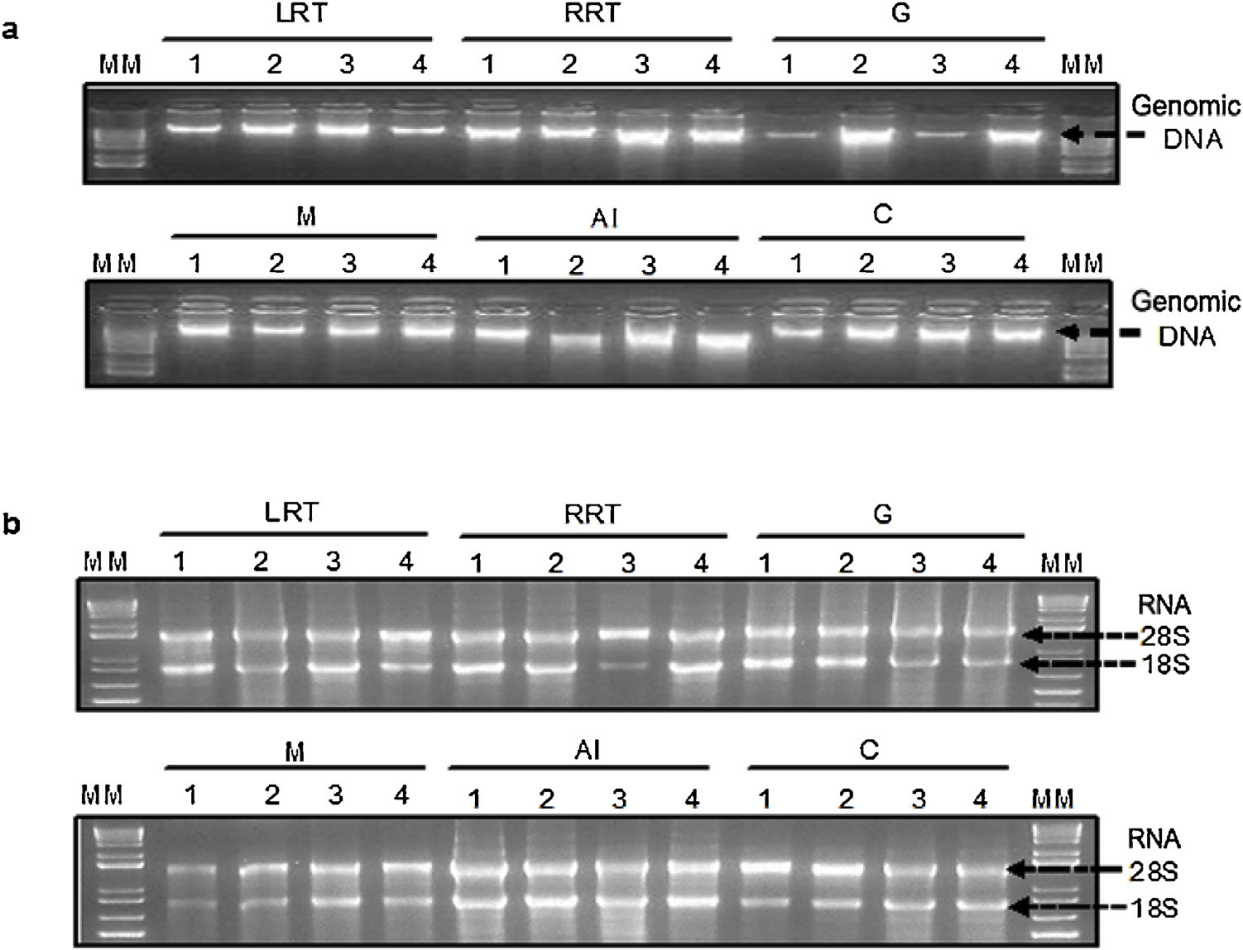
Nucleic acids visualized in an agarose gel 2%, extracted from different tissues of four sea cucumbers. (a) Genomic DNA and (b) total RNA. LRT: Left Respiratory Tree, RRT: Right Respiratory Tree, G: Gonad, M; Muscle, AI: Anterior Intestine and C: Cloaca. The numbers correspond to the dissected organisms. MM: molecular mass marker (1 kb).

The organisms used in this study and the tissues extracted are shown in Fig. 1. With regard to the DNA, a successful extraction was achieved in all the tissues (Fig. 2A) with concentrations registered from 288.2 to 5671.8 ng/μL. The highest concentration of genomic DNA was obtained in the anterior intestine of *I. badionotus* (5671.8 ng/μL per 25 mg of tissue), while the lowest concentrations were registered in the longitudinal muscle bands and the cloaca (269.2 and 288.2 ng/μL per 25 mg of tissue, respectively). The absorbance ratios *A*_260/280 and_ *A*_260/230_ were found in a range between 1.9 and 2.2, which are accepted purity values for molecular studies (Table 1).

**Table 1:** Concentrations of DNA and RNA (ng/μL) obtained from different tissues of sea cucumbers.

Nucleic acid	Tissue	Organism				Absorbance ratios	
		1	2	3	4	260/280	260/230
DNA	LRT	301.3	560	850.2	378.5	2.0 ± 0.02	2.1 ± 0.09
	RRT	703.3	514.3	1384.7	958.1	1.9 ± 0.02	2.1 ± 0.11
	G	479.6	537.9	596.1	420.1	1.9 ± 0.07	2.1 ± 0.01
	M	383.6	269.2	444.9	409.8	2.0 ± 0.03	1.9 ± 0.17
	AI	1251.2	4806.2	5337.0	5671.8	2.0 ± 0.02	2.2 ± 0.03
	C	302.7	676.9	288.2	572.6	2.0 ± 0.03	2.0 ± 0.09
RNA	LRT	798.7	3642.7	1364.3	493.7	2.0 ± 0.03	1.4 ± 0.13
	RRT	1058.4	1731.4	399.6	1619.9	2.0 ± 0.04	1.3 ± 0.30
	G	1030.3	2061.2	3092.4	1570.3	2.0 ± 0.01	1.8 ± 0.09
	M	319.7	464	702.1	599.5	2.0 ± 0.03	0.8 ± 0.21
	AI	4492.6	1651.4	1884.3	814.8	2.0 ± 0.4	1.7 ± 0.21
	C	568.6	521.6	615.5	1318.6	1.8 ± 0.11	1.0 ± 0.25

LRT: Left Respiratory Tree; RRT: Right Respiratory Tree; G: Gonad; M: Muscle; AI: Anterior Intestine; C: Cloaca.

The concentration of obtained RNA using the TRIzol method was higher than 200 ng/μL per 100 mg of tissue, in all tissues analyzed. The integrity of this molecule can be observed by the well-defined bands of the ribosomal subunits 28S and 18S in Fig. 2B.

**Fig. 3:**
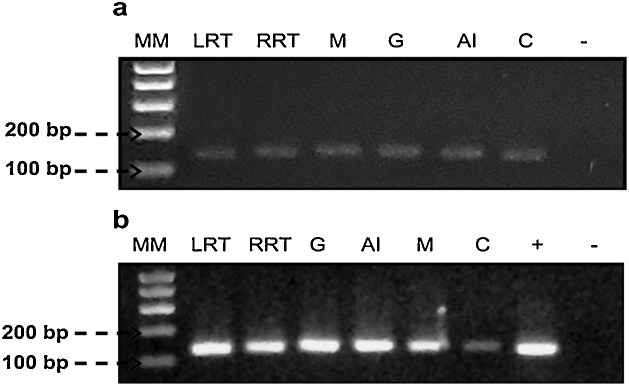
Amplified fragment of ˜150 bp corresponding to 18S rRNA gene visualized in an agarose gel 2%. (a) DNA and (b) cDNA samples. LRT: Left Respiratory Tree, RRT: Right Respiratory Tree, M; Muscle G: Gonad, AI: Anterior Intestine and C: Cloaca. + positive control, − negative control and MM: molecular mass marker (1 kb).

The highest concentration of RNA was obtained in the tissues corresponding to the left respiratory tree, right respiratory tree, gonad, and anterior intestine, with values of up to 4492.6 ng/μL. Concerning the absorbance ratio 260/280 nm, the values were 2.0 in the majority of the tissues, the only exception being the cloaca with an average value of 1.8 ± 0.1. In the case of the ratio 260/230, in the majority of the tissues (respiratory trees left and right, gonad and anterior intestine) values above 1.0 and close to 2.0 were obtained, the only exceptions being the muscle and the cloaca with values of 0.8 and 1.0, respectively (Table 1). From the extracted DNA and the synthesized cDNA, we performed a PCR with specific primers for the 18S rRNA gene. The amplification of a fragment ˜150 bp in a sample of each tissue, suggests that the nuclei acids extracted with both protocols are inhibitors-free (Fig. 3A and B). The concentrations of DNA and RNA obtained in each one of these tissues are adequate for a diversity of analyses at a molecular level. For PCR reaction, 10–20 ng of DNA is required [7], and a higher concentration of 50 ng is necessary for studies of population genetics [8]. For genomic and transcriptomic studies, approximately 3 μg of DNA and RNA are needed. In this sense, the protocol used in the present work allowed obtaining DNA and RNA in sufficient concentration and with good quality to allow completing the studies mentioned above.

The methodologies currently available will form the basis to boost molecular biology research in *I. badionotus;* permitting the analysis of genetic diversity, cloning and gene expression, identification of molecular markers, construction of genetic maps and genome sequencing. In other species of sea cucumbers, such as *Apostichopus japonicus,* the advances in genome sequencing [14] and transcriptome analyses are having a significant impact on the understanding of a diversity of biological mechanisms, such as regeneration, aestivation, and growth [10,15]. These studies will be fundamental to the development of genetic improvement programs in *I. badionotus*.
